# Structural and Functional Changes of the Human Macula during Acute Exposure to High Altitude

**DOI:** 10.1371/journal.pone.0036155

**Published:** 2012-04-30

**Authors:** M. Dominik Fischer, Gabriel Willmann, Andreas Schatz, Kai Schommer, Ahmad Zhour, Eberhart Zrenner, Karl U. Bartz-Schmidt, Florian Gekeler

**Affiliations:** 1 Centre for Ophthalmology, University of Tuebingen, Tuebingen, Germany; 2 Department of Sports Medicine, Medical Clinic, University Hospital Heidelberg, Heidelberg, Germany; Washington University School of Medicine, United States of America

## Abstract

**Background:**

This study aimed to quantify structural and functional changes at the macula during acute exposure to high altitude and to assess their structure/function relationship. This work is related to the Tuebingen High Altitude Ophthalmology (THAO) study.

**Methodology/Principal Findings:**

Spectral domain optical coherence tomography and microperimetry were used to quantify changes of central retinal structure and function in 14 healthy subjects during acute exposure to high altitude (4559 m). High-resolution volume scans and fundus-controlled microperimetry of the posterior pole were performed in addition to best-corrected visual acuity (BCVA) measurements and assessment of acute mountain sickness. Analysis of measurements at altitude vs. baseline revealed increased total retinal thickness (TRT) in all four outer ETDRS grid subfields during acute altitude exposure (TRT_outer_ = 2.80±1.00 μm; mean change±95%CI). This change was inverted towards the inner four subfields (TRT_inner_ = −1.89±0.97 μm) with significant reduction of TRT in the fovea (TRT_foveal_ = −6.62±0.90 μm) at altitude. BCVA revealed no significant difference compared to baseline (0.06±0.08 logMAR). Microperimetry showed stable mean sensitivity in all but the foveal subfield (MS_foveal_ = −1.12±0.68 dB). At baseline recordings before and >2 weeks after high altitude exposure, all subjects showed equal levels with no sign of persisting structural or functional sequels.

**Conclusions/Significance:**

During acute exposure to high altitude central retinal thickness is subject to minor, yet statistically significant changes. These alterations describe a function of eccentricity with an increase in regions with relatively higher retinal nerve fiber content and vascular arcades. However, these changes did not correlate with measures of central retinal function or acute mountain sickness. For the first time a quantitative approach has been used to assess these changes during acute, non-acclimatized high altitude exposure.

## Introduction

Retinal hypoxia is a common pathogenetic mechanism in highly prevalent disorders of vision loss such as diabetic retinopathy, central or branch retinal vein occlusion or less prevalent forms of small vessel disease [1]. Regardless of its upstream etiology, retinal hypoxia upregulates expression of a number of factors including vascular endothelial growth factor (VEGF), which in turn causes increased vascular permeability and downstream retinal edema formation [2]. In line with this concept, increased levels of VEGF have been found in diabetic retinopathy with increased vascular leakage [3], [4]. Furthermore, both supplemental inspired oxygen [5] and pharmacologic inhibition of VEGF [6] have been shown to effectively decrease retinal thickness in existing diabetic macular edema. In venous occlusive disorders, steroids such as triamcinolone have been found to exact their therapeutic effect on reducing/reversing retinal edema formation in part by downregulation of VEGF expression [7]. These findings all support the notion that retinal oxygenation levels play an important role in the maintenance of retinal structure and function and that pathologic levels of hypoxia may result in disruption of the brain-retina-barrier (BRB) with resulting macular edema and disturbances of visual function. This aspect might bear its greatest relevance in the delicate microenvironment of the central retina with its foveal avascular zone and its exceeding importance for key functional tasks such as daylight visual acuity. Indeed, there is robust evidence for changes in central retinal function in response to different oxygenation levels even in healthy subjects both at sea level [8], [9] and during exposure to altitude related hypoxia [10]–[12]. Alterations in central retinal function such as visual acuity, retinal sensitivity and color vision can potentially have deleterious effects especially in mountaineering, where visual orientation, reading instruments and maps can become a life-saving necessity. Detailed quantitative analyses of macular changes at high altitude might be difficult to perform due to unique logistic challenges. Some studies circumvented this problem by recording changes several days after the exposure to altitude [13]–[16]. However, measurements acquired any time after descent may not reflect the changes during acute exposure to high altitude related hypoxia. The research facilities at Capanna Margherita (4559 m, Italy) and readily available helicopter service provide ideal logistical solutions to such an undertaking with the additional benefit of an established ascent profile already used in other studies on high altitude medicine especially in regard to acute mountain sickness [17]–[19]. Our study aimed to precisely quantify changes of central retinal structure and function during acute exposure of healthy volunteers to high altitude using state of the art technology including Spectralis™ HRA+OCT and fundus-controlled microperimetry. In addition these changes were correlated with measures of acute mountain sickness to test a possible causative link.

## Methods

### Study design

**Figure 1:**
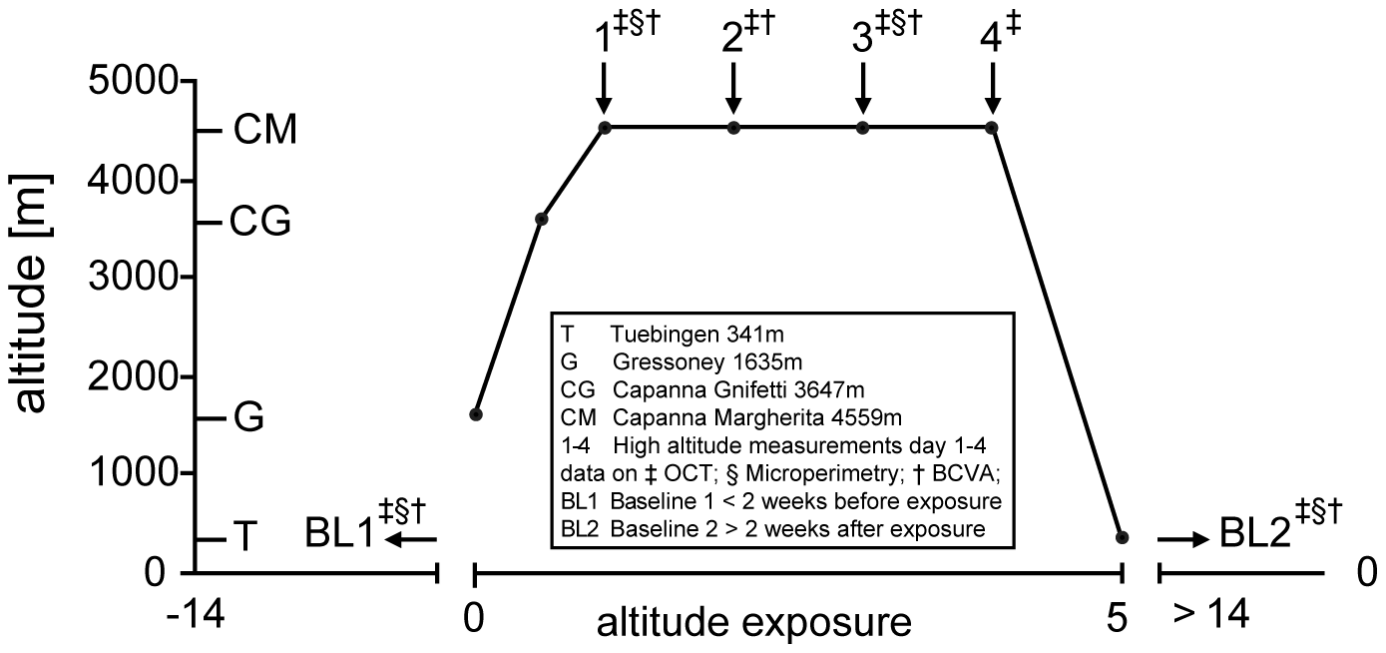
Ascent profile of subjects to the Capanna Margherita (4559 m). Symbols indicate times of measurements performed at high altitude: ^‡^OCT; ^§^Microperimetry; ^†^BCVA; OCT  =  optical coherence tomography; BCVA  =  best corrected visual acuity.

A total of fourteen healthy subjects ascended to the Capanna Margherita (CM; Valais Alps, Italy) according to a previously published ascent profile [19] (Fig. 1): day 0 from Gressoney (Italy) 1635 m to Punta Indren 3260 m by cable car followed by 2 hours (hr) of hiking to the Capanna Gnifetti 3647 m; day 1 ascent to the CM 4559 m in 4–6 hr. All subjects ascended within 24 hr from Gressoney to the CM and spent three nights (from day 1 to day 4) at the CM before descending back to Gressoney on day 4. Before baseline recordings (BL1 =  before and BL2 =  after the climb) and ascent, subjects had to spend >14 days below 2000 m to exclude confounding effects due to previous altitude exposure. All subjects were healthy and physically fit (7 females, 7 males; age 25–54 years). Clinical assessment of acute mountain sickness (AMS) and vital parameters was performed as previously published [19]. Briefly, Lake-Louise (LL) and AMS-cerebral score (AMS-c) of the Environmental Symptom Questionnaire (ESQ III) were used to assess prevalence of AMS defined as LL ≥5 in the presence of headache and AMS-c≥0.70 [20].

Ophthalmological exclusion criteria were presence of disorders affecting the macular region, best corrected visual acuity (BCVA) >0.3 (logMAR) on either eye and/or optical opacities limiting imaging quality. Macular structure was analyzed on all days (BL1, day 1–4 and BL2) in all but one subject: Unfortunately, one male subject had an incomplete data set at BL1 and was therefore excluded from further analysis; all subjects underwent BCVA assessment on BL1, day 1–3 and BL2 and microperimetry was performed on BL1, day 1, day 3 and BL2. The study was performed in accordance with the tenets of the Declaration of Helsinki 1975 (1983 revision) and was approved by the local IRB (Ethik-Kommission der Medizinischen Fakultät und am Universitätsklinikum Tuebingen, IEC project number: 258/2010B01). All subjects gave written informed consent after having been informed of the nature of the research expedition. All equipment was transported by helicopter (Air Zermatt AG Heliport, Zermatt, Switzerland) in airfreight containers provided by the manufacturers.

### Visual Acuity

Freiburg Visual Acuity Test (FrACT, http://michaelbach.de/fract/) was used to measure BCVA according to published guidelines [21], [22]. Briefly, Landolt-C optotypes were displayed on a daily re-calibrated computer monitor (SyncMaster 204B, Samsung Electronics GmbH, Schwallbach, Germany), for self-paced measurement of visual acuity. In this EN ISO 8596 compliant visual test battery, subjects respond by pressing one of 8 buttons (eight alternative forced choice task) to indicate the orientation of the gap in the Landolt-C. The next optotype is presented based on integration of previous responses applying the best Probability Estimation of Sensory Threshold (best PEST) strategy [23]. A total of 18 trials are run to estimate the acuity threshold, which is defined as 56.25% (the middle between 100% and the guessing rate 12.5%).

### Spectral Domain Optical Coherence Tomography

**Figure 2:**
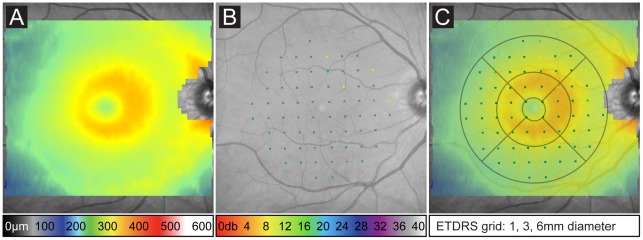
Protocols used to analyze central retinal structure and function in one subject at baseline (BL1). A) Pseudo-colored heatmap diagram indicates total retinal thickness (μm) in a 25×30° volume OCT scan centered on the fovea (color coding scale at bottom). B) Pseudo-colored retinal sensitivity (dB) results in 68 loci within the central 20° projected on the infrared fundus image after co-registration (color coding scale at bottom). C) Multimodal mapping approach of structural/functional data and its analysis in nine distinct ETDRS subfields (dimensions indicated below).

To quantitatively measure the changes of the central retinal tissue two identical Spectralis™ HRA+OCT (Heidelberg Engineering, Heidelberg, Germany) devices were used for the baseline measurements and for the measurements at altitude as previously described [24], [25]. Briefly, pupillary mydriasis was induced in subjects using tropicamide eye drops 5 mg/ml (Mydriaticum Stulln, Pharma Stulln, Stulln, Germany). High-resolution volume scans centered on the fovea were recorded with a scan pattern of 30×25° with 31 B-Scans at 245 μm intervals. To triple signal to noise ratio, 9 scans were averaged while correcting for eye movements using the proprietary TruTrack™ function. To ensure comparability of iterative measurements by correcting for errors such as tilt and orientation, we exported the data from the baseline recordings performed at the University Eye Hospital Tuebingen in the proprietary format (.E2E) and imported it prior to follow-up measurements at altitude. Careful calibration of the laser light source of the Spectralis™ HRA+OCT device was performed after re-assembling at altitude to ensure comparability of measurements. According to the manufacturers instructions (Instructions for the Spectralis Installation Report, Heidelberg Engineering), power of the OCT light source, a superluminescent diode (SLD), was measured at the outlet of the camera using a special adapter provided by Heidelberg Engineering (Sensor PD300, Heidelberg Engineering) and a Ophir powermeter (Nova Powermeter, Spiricon GmbH, Ahrensburg, Germany) set for a test wavelength of 880 nm and range of 3 mW. In the IR/OCT mode, IR-laser was set to 0% laser power using the touch panel service mode and SLD power was measured directly at the objective using the circular ring scan (no laser modulation) at 30° field view. Measurements of SLD laser power both at BL1-2 and at altitude were 1.22 mW. Subsequently, we ensured optical alignment of the spectrometer and compared the SLD spectra in the OCT service screen, which displays a one-line detector output (A-Scan image). Manipulation of the cable loom allowed to “fine tune” the height of the spectrum as this depends to some extent on polarization effects. This provided an almost identical shape of the spectral curve and height of the maximum between both locations of measurement and thus ensured comparability of measurements. Built-in software (Eye Explorer v1.6.4.0 and Spectralis Viewing Module 5.3.2.0) was used to calculate interpolated retinal thickness values (Fig. 2A) using the default macular subfield analysis (ETDRS grid) in reference to the early treatment of diabetic retinopathy study (ETDRS)[26].

### Microperimetry

Fundus controlled microperimetry was performed using two identical Nidek MP1 (NAVIS software version 1.7.4.B1; Nidek Technologies) devices. Chen et al. have previously published test-retest variability, which was applied in this study [27]. Briefly, both pupils were dilated as described above before microperimetry was performed first on the right, then on the left eye while the respective contralateral eye was patched. Microperimetry was conducted in a darkened room. A 2° red cross was used as the fixation target. Real-time correction of involuntary eye movements was achieved by initial co-registration of high-contrast landmarks in the infrared fundus image by the operator. All subjects initially underwent brief training allowing familiarization and practice with correct operation of the response trigger and the stimulus target. This was followed by the first test protocol featuring a white test stimulus of Goldmann III equivalent size (0.4°) with 200 ms duration. Stimulus was presented at 68 loci in a grid covering the central 20° manually centered on the fovea (Fig. 2B). To reduce testing time, a pre-test protocol was programed to present initial 6 dB stimuli at four parafoveal loci. A 4 to 2 staircase strategy was used (as in the following testing protocol) to determine retinal threshold. These values were then used as the starting brightness for testing retinal mean sensitivity (MS) in the remaining, randomly tested loci. The refinement and re-check option was not used.

### Multimodal Mapping

Co-registration of high-resolution OCT volume scans and microperimetry data was performed using a custom-made software tool (MultiModalMapper)[28] as previously published [29]. Briefly, co-registration of the coordinate systems of microperimetry and OCT datasets was based on an affine transformation model, which mapped microperimetry data (source coordinate system) on OCT data (target coordinate system) based on corresponding landmarks manually set on both co-recorded fundus images. After co-registration, the same ETDRS grid algorithm was used to calculate interpolated MS values from test loci within each grid compartment (Fig. 2C). This allowed accurate definition of functional output originating from each grid compartment and thus precise correlation of retinal structure and function in the macular region.

### Statistical Methods

JMP® was used for statistical analysis (Version 8.0.2, SAS Institute, Cary, NC). As measurements of the left and right eye revealed no significant differences (see results section), only the right eye was considered. Furthermore, as measurements at baseline (BL1-2) and at altitude (day 1–4) provided consistent levels respectively, we calculated the mean differences between baseline recordings (BL1-2) and measurements at altitude (mean day 1–4) for a descriptive comparison. Statistical longitudinal analysis was achieved by multivariate analysis of variance (MANOVA) for repeated measures. Data are shown in terms of intra-individual differences (value during exposure – value at BL1) and the 95% confidence limits for each time point. Pearson's correlation coefficient was calculated to evaluate a possible correlation between retinal thickness measures and functional data (MS) per ETDRS grid compartment.

## Results

All 14 subjects successfully ascended to the CM and completed all examinations during 4 days at high altitude as noted in Figure 1. In this cohort, incidence of AMS was 64% on day 1 and prevalence decreased in the following days as expected with acclimatization. Accordingly, AMS-c scores and heart rate reached highest levels on day 1 after arrival at CM, whereas oxygen saturation was expectably decreased (see table 1) with overall lower but non-significant changes in AMS positive subjects.

**Table 1:** Overview of acute mountain sickness (AMS) related parameters.

	BL1	1 late	2 early	2 late	3 early	3 late	4 early	BL2
AMS [%]	0	64	50	29	21	0	14	0
AMS-c [score]	0	1.02±0.65	1.07±0.74	0.70±0.68	0.65±0.81	0.25±0.33	0.33±0.36	0
LL [score]	0	5.36±2.21	5.36±2.59	3.93±2.09	4.00±3.37	2.07±1.54	2.43±2.14	0
Heart rate [min-1]	60.4±7.35	88.43±5.97	83.43±10.12	82.71±9.45	77.14±12.18	75.22±16.60	73.64±13.43	58.14±6.92
SpO2 [%]	98.50±1.34	69.36±4.36	72.14±5.86	74.43±7.07	73.93±6.04	76.78±3.83	79.36±4.31	98.29±1.27

data are mean±standard deviation; n = 14; AMS-c  =  acute mountain sickness cerebral score; LL  =  Lake Louise; SpO2  =  oxygen saturation by pulse oximetry; late  =  c. 21 h; early  =  c. 6 h.

Statistical analysis of all data sets on retinal structure and function showed no statistical difference between baseline recordings before and after acute exposure to high altitude (BL1 vs. BL2), thus indicating the stability of our baseline recordings and the reversibility of any changes associated with high altitude exposure. Furthermore, there were no statistically significant differences between data from the right and the left eyes of subjects. Therefore, we decided to consider only data from right eyes as a rather conservative measure to minimize false positive findings.

**Figure 3:**
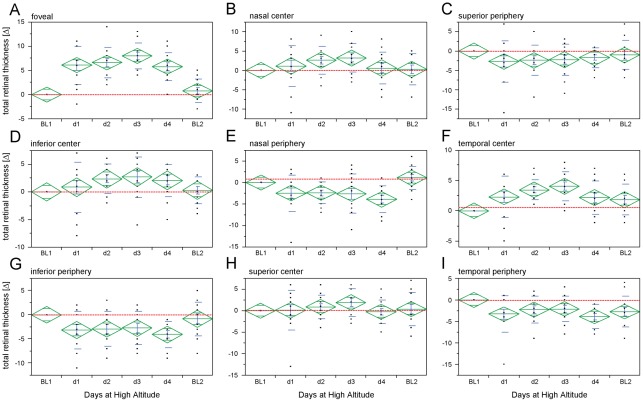
Structural changes at the posterior pole during acute exposure to high altitude in healthy subjects. A–I) Intraindividual changes (expressed as differences: baseline-altitude) of total retinal thickness within ETDRS subfields show decreased total retinal thickness (TRT) in the foveal and inner subfields and an increased TRT in outer subfields. Red dashed lines indicate BL1 level; center of diamonds represent mean, top and bottom horizontal lines indicate the 95% confidence interval, whiskers indicate standard error and horizontal lines display standard deviation; n = 13.

SD-OCT volume scans of the central retina showed significant changes with a gradient from the foveal center to the more peripheral areas of the macula. While the foveal ETDRS subfield demonstrated a noteworthy reduction of TRT at altitude (mean of day 1–4) compared to baseline recordings (TRT_foveal_ = –6.62±0.90 μm; mean±95%CI), the neighboring inner ETDRS subfields showed only minimal changes (TRT_inner_ = –1.89±1.93 μm). In contrast, TRT was increased in the outer ETDRS subfields (TRT_outer_ = 2.80±1.99 μm) at altitude vs. baseline. Statistical longitudinal analysis (MANOVA) reflected the difference between recordings at baseline and altitude and demonstrated rather stable readings at both settings, i.e. there was only limited variability during day 1–4 and between measurements at BL1 vs. 2 (Fig. 3, data are summarized in table 2).

**Table 2:** Changes (intra-individual differences) of total retinal thickness (TRT) in all ETDRS subfields at all time-points.

	foveal	outer-nasal	inner-nasal	outer-superior	inner-superior	outer-temporal	inner-temporal	outer-inferior	inner-inferior
**BL1**	0.00	0.00	0.00	0.00	0.00	0.00	0.00	0.00	0.00
**day1**	–6.08±2.38	2.46±2.56	–1.15±3.19	2.69±3.23	–0.08±2.80	3.31±2.57	–2.31±2.09	3.15±2.33	–0.85±2.74
**day2**	–6.62±1.91	2.38±1.52	–2.62±2.18	2.38±2.34	–0.85±1.67	2.23±1.91	–3.46±1.01	2.92±2.19	–2.38±1.63
**day3**	–8.00±1.64	2.62±2.76	–3.23±2.22	2.23±2.47	–1.92±1.97	2.08±1.77	–4.08±1.45	2.77±2.08	–2.69±2.23
**day4**	–5.77±1.75	3.92±1.89	–0.62±2.51	1.69±1.57	0.23±1.66	3.85±1.69	–2.15±1.67	4.08±1.65	–2.08±1.77
**BL2**	–0.77±1.48	–1.08±1.65	–0.31±2.44	1.00±2.27	–0.38±2.35	2.69±2.19	–1.85±1.54	0.85±2.12	–0.31±1.47

Longitudinal intra-individual changes of total retinal thickness in all nine ETDRS subfields at each time-point as difference from baseline (BL1).

ETDRS  =  early treatment of diabetic retinopathy; BL =  baseline; data are displayed as mean±95% confidence interval in μm; n = 13.

**Figure 4:**
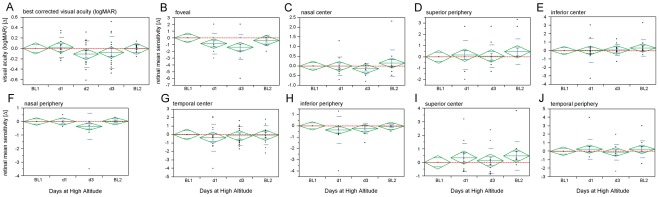
Functional changes at the retinal center during acute exposure to high altitude in healthy subjects. A) Intraindividual changes (expressed as differences: baseline-altitude) of best corrected visual acuity (BCVA in logMAR) and B–J) retinal mean sensitivity (MS in dB) values for each ETDRS subfield show no significant changes except in the foveal subfield on day 3 but not on day 1. Red dashed lines indicate BL1 level; center of diamonds represent mean, top and bottom horizontal lines indicate the 95% confidence interval, whiskers indicate standard error and horizontal lines display standard deviation; n = 14.

Functional test results displayed stable performance throughout the study with no significant mean differences in BCVA measurements between recordings at baseline and altitude (logMAR  = –0.06±0.08 dB, detailed longitudinal data are displayed in Fig. 4A). Topographic analysis of retinal sensitivity was performed using the fundus-controlled microperimetry on day 1 and day 3 at high altitude. In line with stable BCVA measurements, all ETDRS grid subfields maintained stable mean retinal MS compared to baseline values. Statistical longitudinal analysis (Fig. 4B–C) relvealed one exception regarding the foveal subfield at day3 (MS_foveal_ = –1.38±1.12 dB) but not on day 1 (MS_foveal_ = –0.85±0.92 dB). The full functional data set is summarized in table 3 and table 4.

**Table 3:** Changes (intra-individual differences) of retinal mean sensitivity (MS) values in all ETDRS subfields at all time-points measured.

	foveal	outer-nasal	inner-nasal	outer-superior	inner-superior	outer-temporal	inner-temporal	outer-inferior	inner-inferior
**BL1**	0.00	0.00	0.00	0.00	0.00	0.00	0.00	0.00	0.00
**day1**	–0.85±0.92	0.01±0.11	–0.02±0.29	0.16±0.78	0.35±0.64	0.24±0.71	–0.36±0.97	–0.37±0.72	0.03±0.84
**day3**	–1.38±1.12	–0.35±0.59	–0.15±0.17	0.07±0.60	0.11±0.56	–0.09±0.57	–0.17±0.67	–0.22±0.28	0.08±0.39
**BL2**	–0.38±0.47	0.02±0.05	0.15±0.42	0.46±0.69	0.48±0.64	0.23±0.60	–0.08±0.69	–0.04±0.09	0.32±0.59

Longitudinal intra-individual changes of retinal mean sensitivity (MS) in all nine ETDRS subfields at each time-point as difference from baseline (BL1).

ETDRS  =  early treatment of diabetic retinopathy; BL =  baseline; data are displayed as mean±standard deviation in dB; n = 14.

**Table 4:** Changes (intra-individual differences) of BCVA values in all ETDRS subfields at all time-points measured.

	decimal	logMAR
**BL1**	0.00	0.00
**day1**	–0.05±0.19	0.02±0.11
**day2**	0.17±0.20	–0.11±0.15
**day3**	0.11±0.26	–0.08±0.18
**BL2**	0.01±0.06	–0.01±0.05

Longitudinal intra-individual changes of best corrected visual acuity at each time-point as difference from baseline (BL1).

logMAR  =  log of minimal angle of resolution; n = 14.

Co-registration of the structural and functional data in the MultiModalMapper software allowed testing for topographically related association of structural and functional changes at altitude. Pearson's correlation analysis between MS and TRT values from foveal, inner and outer ETDRS subfields showed no statistical significant association (*p* >0.05, see table S1).

As previous studies have provided equivocal results regarding a correlation between AMS and ocular changes under hypoxia and/or at high altitude [19], [30]–[35], we calculated the association between AMS-c, heart rate or SpO2 and changes of retinal structure (TRT) in foveal, inner or outer ETDRS subfields. There was no significant correlation between TRT values of any subfield and AMS-c (*p* >0.05). For clinical parameters no significant correlation was found for heart rate (*p* >0.05). Only SpO2 correlated significantly with TRT values of the foveal (r = 0.61, *p* = 0.03) and inner (r = 0.66, *p* = 0.01), but not the outer subfields (see table S2).

## Discussion

This study was undertaken to objectively quantify structural and functional changes in the central retina during acute high altitude exposure as hypoxic challenge. The human macula is a highly specialized region at the posterior pole of ca. 3 mm diameter (foveal and inner ETDRS subfields), which relays crucial visual information such as daylight visual acuity. It features some anatomic peculiarities compared to the rest of the retina, which convey increased optical resolution peaking in the rod free foveola. Most notably, retinal vessels and nerve fiber bundles circumvent the macula in superior and inferior arcades to minimize light scatter in the center (outer ETDRS subfields). The central foveal region is completely avascular and thus dependent on diffusion of oxygen and other nutrients from the choroidal blood flow (outer retina) and a surrounding capillary network (inner retina) [36]. As a result, especially its inner retinal neurons are prone to ischemic conditions and there is abundant evidence for secondary structural and functional changes in the macula e.g. due to diabetic small vessel disease, smoking or other factors, which affect oxygen transport to this avascular neuronal tissue [1], [5], [8], [9]. This is also reflected by the definition of clinically significant macular edema (CSME) [26], which calls for retinal thickening and/or hard exudates with adjacent retinal thickening within 500 µm of the macular center (foveal ETDRS subfield) or one or more disc diameters of retinal thickening, part of which needs to be within one disc diameter of the macular center (inner ETDRS subfields).

As acute exposure to high altitude was expected to challenge this delicate microenvironment in the foveal avascular zone, we initially speculated on a hypoxia mediated increase in central retinal thickness, possibly with some reduction of functional measures. However, while changes in TRT during acute exposure to high altitude related hypoxia demonstrated robust significance levels especially in the foveal and outer ETDRS subfields, this was largely due to the exceptionally low test-retest variability of the Spectralis™ HRA+OCT [37], [38]. The limited relative change of TRT at altitude in all ETDRS subfields clearly demonstrates, that acute exposure to 4559 m does not lead to CSME. This indicates a superior dynamic range of autoregulatory counter mechanisms within the macular region to meet the demands of oxygenation even under conditions of acute exposure to high altitude related hypoxia in healthy subjects. However, exposure to higher altitudes and/or longer duration of altitude related hypoxia may have a more deleterious effect and can not be ruled out by this study. Interestingly, outer ETDRS subfields showed limited, but significant increase in TRT. This paramacular area features an inherently higher relative content of retinal nerve fiber bundles and retinal vessels. As previous studies at very high (ca. 8000 m) and high altitude (ca. 4500 m) have provided evidence for increased thickness of retinal nerve fibers and dilated, tortuous retinal vessels, it seems plausible to assume this as a contributing factor for the relative increase in TRT in the outer ETDRS subfields [13], [19], [30], [33]. One previous OCT study on retinal changes associated with high altitude exposure and acute mountain sickness showed no significant alteration in any of the ETDRS subfields [13]. However, subjects in this study were examined at an altitude of 247 m two weeks before and after returning from the expeditions in the Himalayas. Given the minute absolute changes of retinal thickness to be expected, we argue that lack of significant findings in this previous study might be attributed to its study design. In fact, our baseline recordings were recorded similarly, i.e. ≥ two weeks prior and after acute exposure to high altitude and showed no statistical significant difference. In contrast, recordings at altitude provided evidence for small, yet statistically significant changes of TRT in ETDRS subfields when compared to these baseline recordings. Correlation analyses between TRT changes in ETDRS subfields and AMS-c showed no significant association. Of clinical parameters associated with AMS severity, only SpO2 demonstrated a significant correlation with TRT of foveal and macular but not paramacular subfields. This lack of robust association between ocular changes and AMS parameters is in line with previous observations that AMS occurs independently of optic disc edema formation [19], but contrasts reports on significant correlations between AMS vs. optic disc swelling, increased optic nerve sheath diameter, increased corneal thickness or increased retinal capillary blood flow [30], [31], [33], [34]. Part of the discrepancy can be explained by differences in ascent profiles, absolute altitude, duration of altitude exposure, methods of data acquisition (e.g. qualitative vs. quantitative) and examination protocol (i.e. recording at altitude vs. after descent). Here, we see a major strength of our study design, which used an established ascent profile and relied on state of the art quantitative diagnostic instruments, which are routinely used in clinical ophthalmology. This is intended to facilitate comparability with future studies and help to relate the observed changes with clinical findings in prevalent pathologic ischemic conditions. Our study design relied on the known and exceptionally low test-retest variability of the Spectralis™ HRA+OCT [37], [38]. But even though all our data – including published results in this Journal [19] – point towards the notion that all structural changes in the eye are completely reversible, iterative recordings before exposure (BL1) rather then comparison of baseline recordings (BL1 vs. BL2) would have additionally strengthened the repeatability assessment in this study. The nature of the study resulted in different recording environments such as differences in air composition between baseline measurements and measurements at high altitude. As SD-OCT measurements in such an environment have never been performed before, systemic errors can not entirely be ruled out. However, the bidirectional difference (i.e. decrease in the foveal subfield vs. increase in extrafoveal subfields) observed make this unlikely.

In conclusion our present findings indicate that acute exposure to high altitude related hypoxia does not result in macular edema, but slight increase in perimacular retinal thickness. These changes are not associated with changes of central retinal function as assessed by BCVA and microperimetry and are fully reversible after descent to lower altitudes. However, with the exception of the foveal central subfield, the thickness changes at high altitude in the other subfields are very small and not much different than the differences between Baselines 1 and 2. Therefore, clinical and/or biological significance of these findings has to be critically assessed. For example, it is unclear how such a hypoxic challenge would affect subjects with existing pathologic changes such as diabetic retinopathy or how these structures would react to even higher altitude e.g. during exposure in the Himalayas. Further studies might be able to define and even predict the associated risk e.g. for diabetic patients to acute exposure of high altitude related hypoxia.

## Supporting Information

Table S1 **Correlation analysis between retinal mean sensitivity (MS) and total retinal thickness (TRT) values from foveal, inner and outer ETDRS subfields.**
(DOCX)

Table S2 **Correlation analysis between acute mountain sickness (AMS) parameters and total retinal thickness (TRT) values from foveal, inner and outer ETDRS subfields.**
(DOCX)
