# Supplementary material for: Structural and Functional Changes of the Human Macula during Acute Exposure to High Altitude
Source: PLoS One. 2012 Apr 30;7(4):e36155. doi: 10.1371/journal.pone.0036155 (PMC3340355; doi:10.1371/journal.pone.0036155)
Supplement: Table S1 — Correlation analysis between retinal mean sensitivity (MS) and total retinal thickness (TRT) values from foveal, inner and outer ETDRS subfields. (DOCX) [file pone.0036155.s001.docx]

**Table S1**

| **Correlation analysis between retinal mean sensitivity (MS) and total retinal thickness (TRT) values from foveal, inner and outer ETDRS subfields on day3** | | | |
| --- | --- | --- | --- |
|  | **foveal TRT** | **inner TRT** | **outer TRT** |
| **foveal MS** | r = 0.53; *p* = 0.06 | - | - |
| **inner MS** | - | r = 0.42; *p* = 0.15 | - |
| **outer MS** | - | - | r = -0.08; *p* = 0.80 |
|  |  |  |  |
| ETDRS = early treatment of diabetic retinopathy; n = 14. | | | |
