# Supplementary material for: Structural and Functional Changes of the Human Macula during Acute Exposure to High Altitude
Source: PLoS One. 2012 Apr 30;7(4):e36155. doi: 10.1371/journal.pone.0036155 (PMC3340355; doi:10.1371/journal.pone.0036155)
Supplement: Table S2 — Correlation analysis between acute mountain sickness (AMS) parameters and total retinal thickness (TRT) values from foveal, inner and outer ETDRS subfields. (DOCX) [file pone.0036155.s002.docx]

**Table S2**

| **Correlation analysis between acute mountain sickness (AMS) parameters and total retinal thickness (TRT) values from foveal, inner and outer ETDRS subfields on day3** | | | |
| --- | --- | --- | --- |
|  | **foveal TRT** | **inner TRT** | **outer TRT** |
| **AMS-c [score]** | r = 0.16; *p* = 0.59 | r = 0.07; *p* = 0.82 | r = -0.43; *p* = 0.14 |
| **Heart rate [min-1]** | r = -0.17; *p* = 0.57 | r = 0.18; *p* = 0.57 | r = 0.20; *p* = 0.51 |
| **SpO2 [%]** | r = 0.61; *p* = 0.03 | r = 0.66; *p* = 0.01 | r = 0.55; *p* = 0.05 |
|  |  |  |  |
| ETDRS = early treatment of diabetic retinopathy; n = 14. | | | |
